# Comparative matched-pair cohort analysis of the short-term clinical outcomes of mesenchymal stem cells versus hyaluronic acid treatments through intra-articular injections for knee osteoarthritis

**DOI:** 10.1186/s40634-020-00310-1

**Published:** 2020-11-13

**Authors:** Yong Sang Kim, Dong Suk Suh, Dae Hyun Tak, Pill Ku Chung, Yoo Beom Kwon, Tae Yong Kim, Yong Gon Koh

**Affiliations:** Department of Orthopaedic Surgery, Center for Stem Cell & Arthritis Research, Yonsei Sarang Hospital, 10, Hyoryeong-ro, Seocho-gu, Seoul, Republic of Korea

**Keywords:** Mesenchymal stem cells, Hyaluronic acid, Osteoarthritis, Knee, Injection

## Abstract

**Purpose:**

Intra-articular injection of hyaluronic acid (HA) has shown promises in reducing pain and improving physical function in knee osteoarthritis (OA). Recently, cell-based therapies using mesenchymal stem cells (MSCs) have emerged as potential treatments. However, few studies have compared the treatment outcomes between MSCs and HA. This study aimed to compare the clinical and radiological outcomes of intra-articular injections of MSCs versus HA in patients with knee OA.

**Methods:**

A cohort of 209 patients with knee OA were retrospectively screened for those who underwent intra-articular injections using MSCs or HA. Thirty MSC-treated patients (MSC group) were pair-matched with thirty HA-treated patients (HA group) based on gender and age. Clinical outcomes were evaluated using the visual analog scale (VAS), International Knee Documentation Committee (IKDC) rating system, and Lysholm scoring system. Radiological evaluation was assessed using the Kellgren-Lawrence (K-L) grading system.

**Results:**

MSC treatment yielded consistent significant improvements in VAS, IKDC and Lysholm scores. In the HA group, VAS scores significantly decreased at 1 month, slightly increased at 3 months, and increased significantly from 3 months to 1 year after injection. The IKDC and Lysholm scores improved significantly until 3 months, but gradually worsened thereafter. Significantly greater improvements in VAS (*P* = 0.041), IKDC (*P* = 0.014), and Lysholm (*P* = 0.020) scores were observed in the MSC group compared to those in the HA group at 1-year post-treatment. The K-L grade worsened in a few patients, especially those in the HA group, albeit no significant difference.

**Conclusions:**

MSC group showed better VAS, IKDC, and Lysholm scores at 1-year post-treatment, compared to the HA group, although earlier clinical improvements were superior in the HA group for the initial 3 months.

**Level of Evidence:**

Therapeutic study, Level III.

## Background

Osteoarthritis (OA) is a prevalent joint disease whose main pathological feature is a chronic cycle of aberrant attempts to repair the implicated joints, thereby leading to inflammation and tissue degradation [37]. The knee is the principal peripheral joint affected, resulting in pain, stiffness, and a progressive loss of function [8]. According to the OA Research Society International, OA management should reduce pain and inflammation, slow down cartilage degradation, improve function, and reduce disability [13]. Treatment can be broadly classified into surgical and non-surgical treatments, and non-surgical treatments include intra-articular injections, physical modalities, alternative therapies, oral analgesics, and the reduction of modifiable risk factors [2]. Among these, intra-articular injections have been widely used over the past few decades because they present a low risk of complications while providing potential pain relief and improving physical function [57]. Intra-articular injections of hyaluronic acid (HA), a natural glycosaminoglycan found in the synovial fluid which acts as a lubricant and an elastic shock absorber during joint movements [38], can potentially restore the effects of the synovial fluid to protect against cartilage erosion and reduce synovial inflammation [16, 38]. Several studies have demonstrated its beneficial effects, including pain reduction, knee function improvement, and delayed need for arthroplasty [10, 19, 41]. However, the therapeutic effect of HA injections is not permanent [29, 47].

Recently, mesenchymal stem cells (MSCs) have been proposed to be used for cell-based therapies for OA due to their immunomodulatory properties [21, 32]. Considering that the pathogenesis of OA is based on degeneration and inflammation, the therapeutic properties of MSCs, including paracrine [4, 20], anti-inflammatory [56], and immunomodulatory effects [60], could contribute towards restoring the intra-articular environment [43]. While several studies have reported improvements in clinical outcomes following MSC injection in patients suffering from knee OA [11, 23, 62], only a few studies have compared its clinical outcomes with those of HA injection [35, 55]. Nonetheless, the MSCs used in these studies were derived from the peripheral blood [55], umbilical cord [35], and bone marrow [35]. Therefore, this study aimed to compare the clinical and radiological outcomes of intra-articular injections of adipose-derived MSCs versus HA in patients with knee OA. We hypothesized that patients who received MSC injections would have better outcomes than those treated with HA injection.

## Methods

### Patient selection and study design

**Fig. 1:**
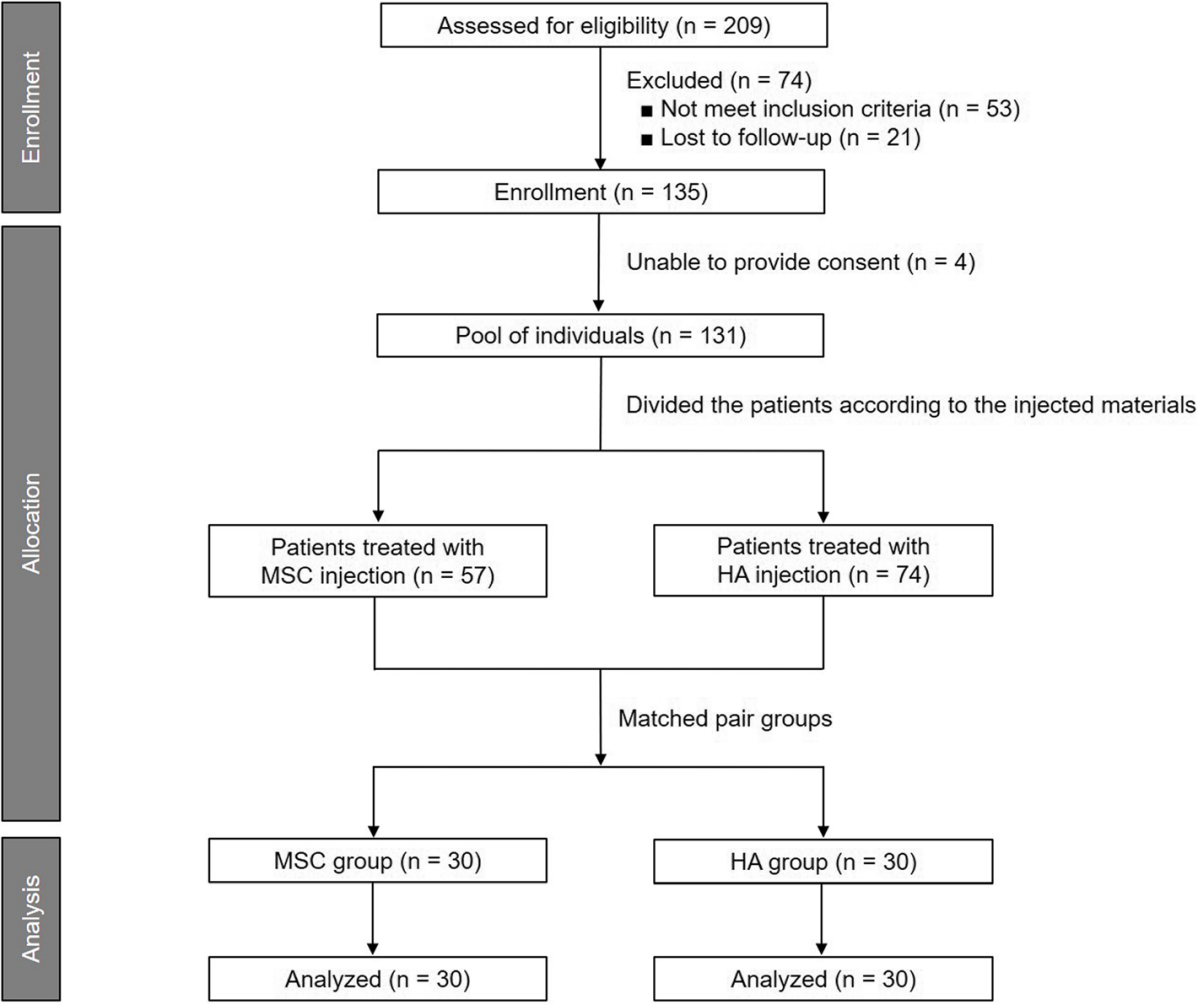
CONSORT flow diagram depicting the patient enrollment process for this study. MSC, mesenchymal stem cell; HA, hyaluronic acid

This study was reviewed and approved by the Institutional Review Board of Yonsei Sarang Hospital (registration number 19-E003–004), and written informed consent was obtained from all participants. We retrospectively reviewed the medical records of 209 consecutive patients with knee OA who were treated with intra-articular injections of MSCs or HA at our clinic and had completed 1 year of follow-up between October 2010 and September 2017. The inclusion criteria were knee OA confirmed by clinical evaluation, radiography, and magnetic resonance imaging (MRI); and symptoms of unilateral knee joint pain and/or functional limitations despite a minimum of 3 months treatment with oral non-steroidal anti-inflammatory drugs. The exclusion criteria were a previous history of HA and/or steroid injection within 1 year; comorbidities in hip or ankle joints; or hematological or cardiovascular disease(s), systemic infection(s), or immunosuppressive disorder(s). Patients who had knee instability, varus or valgus malalignment of the knee joint of ≥5°, metabolic arthritis, joint infections, or large meniscal tears were also excluded. Based on the inclusion and exclusion criteria, 135 patients (MSC injection: 60, HA injection: 75) were eligible. Four of them declined consent and 131 patients were enrolled. After a matching process, 60 patients were finally chosen (Fig. 1). Thirty patients treated with an MSCs were identified and assigned to the MSC group. Thirty patients treated with HA were then matched to the MSC-treated patients, based on gender first, followed by age. The matching process was performed by an independent scientist blinded to the patient’s identity, medical history, and clinical complaints, to eliminate potential sources of bias. The minimum follow-up was 12 months for all patients.

### Collection of subcutaneous adipose tissue

Sample collection and MSC isolation were performed as described previously [30]. Subcutaneous adipose tissue samples were obtained through tumescent liposuction from the gluteal regions of the patients 1 day before MSC injection. We aimed to routinely collect 140 mL of adipose tissue, of which 120 mL was used for the injection, and 20 mL was subjected to laboratory analysis to examine the plastic-adherent cells that form colony-forming unit fibroblast (CFU-F) and confirm the multilineage differentiation of adipose-derived stem cells.

### Isolation of stromal vascular fraction and MSCs from subcutaneous adipose tissue

In the operating room, the aspirated adipose tissue (120 mL) was suspended in phosphate-buffered saline solution before being placed in a sterile box and transported to the laboratory. Mature adipocytes and connective tissues were separated from the stromal vascular fraction (SVF) by centrifugation [65]. Prior to insertion, bacteriologic tests were performed to ensure the absence of contamination in the samples, and the viability of cells was assessed using the methylene blue dye exclusion test. The remaining 20 mL of adipose tissue was processed by the same method and used for cell analysis.

### Assessment of plastic-adherent cells that form CFU-F and immunophenotyping of adipose-derived stem cells

MSCs were originally referred to as fibroblastoid colony-forming-cells because one of their characteristic features is adherence to tissue culture plastic and generation of colonies when plated at low densities [14, 45]. The CFU-F assay were examined to confirm the ability to generate mesenchymal progenitors of the adipose-derived stem cells. The cells were cultured in T25 flasks at a final concentration of 16 cells/cm^2^ to evaluate the frequency of mesenchymal-like progenitors. Colonies of ≥50-cell aggregates were scored under an optical microscope to assess the ability to form colonies. Cells regularly seeded at 50 cells/cm^2^ were expanded till reaching the adequate number for analyzing the flow cytometric immunophenotype using fluorescence-activated cell sorting (FACS). MSC marker phenotyping was performed using CD14, CD34, CD90, and CD105 antibodies according to an established protocol [9, 49]. For analysis of the flow cytometric immunophenotype using FACS, 2 × 10^6^ of cells are required per a CD marker. Therefore, we obtained 8 × 10^6^ of cells for four CD markers by culture expansion process.

### Confirmation of multilineage differentiation of adipose-derived stem cells

Adipose-derived stem cells were plated at 5 × 10^3^ cells/cm^2^ in Dulbecco’s modified Eagle’s medium (Hyclone, Logan, UT) supplemented with 10% fetal bovine serum (Hyclone, Logan, UT), and allowed to adhere for 24 h. The culture medium was then replaced with specific inductive media in order to determine the adipogenic, osteogenic, and chondrogenic differentiation potential [34]. We evaluated the capacity of the human subcutaneous adipose tissue to generate mesenchymal progenitors according to CFU-F.

### Injection of mesenchymal stem cells or hyaluronic acid

All injections in both groups were performed using the same technique by an experienced senior orthopedic surgeon. Patients lay down on the table in a supine position with their knees extended during intra-articular injection. An arthrocentesis was performed to eliminate a knee effusion, before MSC or HA were administered by transversely inserting a needle between the articular surface and patellofemoral joint in the midpoint of the patella, after pushing the patella upwards and shifting it to the lateral side [52]. 3.0 mL of sodium hyaluronate [20 mg/mL, molecular weight: 6 × 10^6^ Da (Da)] was used for HA injection. The patients were advised against additional treatments including physical therapy, acupuncture, steroid injection, and opioid or strong analgesics until 1 year after the injection. They were also told to avoid weight-bearing motions that impose an excessive burden on the affected knee, such as standing for prolonged periods, jogging, and lifting heavy objects, for the first 3 days.

### Outcome assessments

All patients were evaluated clinically and radiologically before injection and during follow-up(s). For clinical evaluation, the visual analog scale (VAS), International Knee Documentation Committee (IKDC) [22], and Lysholm [31] scores were collected. Adverse events were recorded for safety evaluation. Radiological evaluations included a weight-bearing anterior-posterior (AP) view, true lateral view at 30° of knee flexion, and hip-to-ankle standing AP radiograph on a long cassette. To avoid potential bias, an independent observer, who was a musculoskeletal-trained radiologist not involved in the care of patients and blinded to the intention of this study, performed the radiological evaluation. The Kellgren-Lawrence (K-L) grading system [25] was used to assess the AP radiographs.

### Statistical analysis

The matching process was performed based on gender and age using nearest neighbor matching. To determine the necessary power for our study, an a priori power analysis based on IKDC was performed. Based on our a priori power analysis to obtain a power of 0.80 or higher with a ratio of 1:1, we need a minimum of 30 patients in each group. The primary dependent variables were VAS, IKDC, and Lysholm scores as clinical outcomes, and K-L grade as radiological outcome. Descriptive statistics were calculated as mean ± standard deviation for continuous variables and frequencies and proportions for categorical variables. The Wilcoxon signed-rank test was performed to compare the pre- and post-operative clinical values over the follow-up period, while the Mann-Whitney *U* test was used to compare the results between the two groups. The Fisher exact test was used to compare the categorical data. Statistical analyses were performed using SPSS (v13.0; IBM Corp., Armonk, NY, USA), with a *P* value < 0.05 being considered as statistically significant.

## Results

### General characteristics

The final study population included 22 men and 38 women with a mean age of 63.1 years (range: 56–71 years). There was no significant difference for body mass index, side of involvement, follow-up period, and K-L grade between the groups (Table 1). No clinically significant adverse event was noted during the 1-year follow-up period. Although mild swelling of knee joints was observed in three cases (MSC group: 2, HA group: 1), it was resolved without intervention. Subcutaneous induration was observed at the fat harvest site at the buttock area in three cases in the MSC group; however, it was also resolved without intervention by the 6-months follow-up.

**Table 1:** Comparison of baseline demographics in the study groups

	MSC (*n* = 30)	HA (*n* = 30)	*P* value
Age, years	63.0 ± 3.2 (57–70)	63.2 ± 3.8 (56–71)	0.769
Gender, male/female, n	11/19	11/19	> 0.999
Body mass index, kg/m^2^	26.4 ± 1.5 (22.9–28.9)	26.6 ± 1.5 (24.5–29.5)	0.546
Side of involvement, right/left, n	14/16	18/12	0.309
Follow-up period, months	14.2 ± 3.4 (12–24)	15.2 ± 3.9 (12–25)	0.293
Kellgren-Lawrence grade, n (%)			0.769
1	2 (6.7)	3 (10.0)	
2	10 (33.3)	9 (30.0)	
3	12 (40.0)	13 (43.3)	
4	6 (20.0)	5 (16.7)	

Data are expressed as mean ± standard deviation (range) unless otherwise indicated. *MSC* Mesenchymal stem cell, *HA* Hyaluronic acid

### Isolation and characterization of cells

The isolation and characterization procedures determined that adipose-derived stem cells made up 9.4% (range: 8.5–11.3%) of the SVF. Consequently, an average of 7.6 × 10^7^ cells in the SVF, which contained an average of 7.1 × 10^6^ stem cells (range: 6.5 × 10^6^–8.6 × 10^6^ cells), were used for MSC injection. FACS characterization indicated positive expressions of CD90 (99.17%) and CD105 (94.62%), and negative expressions of CD34 (5.34%) and CD14 (2.64%). The treated stem cells exhibited adipogenic, osteogenic, and chondrogenic differentiation potentials after staining.

### Clinical outcomes

**Fig. 2:**
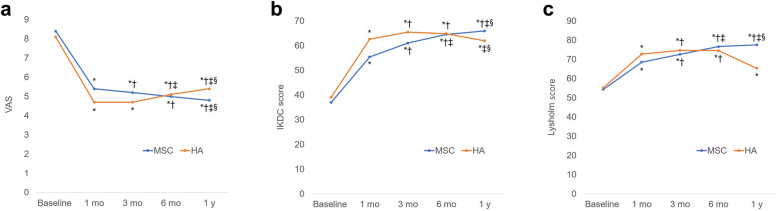
Graphs demonstrating the changes in the **a** visual analog scale (VAS), **b** International Knee Documentation Committee (IKDC), and **c** Lysholm scores. Significantly different from ^*^baseline, ^†^1-month follow-up, ^‡^3-month follow-up, ^§^6-month follow-up

The mean VAS, IKDC, and Lysholm scores at baseline and at 1-month, 3-months, 6-months, and 1-year follow-ups are summarized in Table 2. The mean VAS score in the MSC group significantly and progressively decreased until 1-year post-treatment as compared to baseline (*P* <  0.05 for all; Fig. 2a). On the contrary, the mean VAS score in the HA group significantly decreased at 1 month post-injection with reference to the baseline (*P* <  0.05), slightly increased at the 3-month follow-up (*P* = 0.317), and gradually increased significantly from 3 months to 1-year post-injection (*P* <  0.05) (Fig. 2a). A significantly greater pain relief was reported in the MSC group compared to that in the HA group at the 1-year follow-up (*P* = 0.041; Table 2). The mean IKDC and Lyshlom scores in both groups showed similar trends over the follow-up period. In the MSC group, the mean IKDC and Lysholm scores were significantly improved at 1 month after injection as compared to the baselines (*P* <  0.001 for both). Further significant improvements in the mean IKDC and Lysholm scores were observed until 1-year post-treatment (*P* <  0.05 for all; Fig. 2b and c). In the HA group, the mean IKDC and Lysholm scores were also significantly improved at 1-month and 3-months post-injection as compared to the baseline (*P* <  0.001 for all; Fig. 2b and c). However, the mean IKDC and Lysholm scores gradually decreased from 3-months to 1-year post-injection, albeit no significant difference (*P* > 0.05 for all; Fig. 2b and c) with the exception of the IKDC score at 1-year post-injection with reference to that at 6-months post-injection (*P* = 0.001; Fig. 2b). There were significant differences in the IKDC scores at 1-month, 6-months, and 1-year, and in the Lysholm scores at 1-month and 1-year post-injection between the groups (Table 2). Although more pronounced clinical improvements were evident in the HA group during the early follow-up periods, the MSC group eventually exhibited superior clinical improvements at the 1-year follow-up.

**Table 2:** Comparison of preoperative and postoperative clinical outcomes

	MSC	HA	*P* value
VAS			
Baseline	8.4 ± 1.1	8.1 ± 1.1	0.346
1 month	5.4 ± 1.4	4.7 ± 1.0	0.039
3 months	5.2 ± 1.3	4.7 ± 1.1	0.111
6 months	5.0 ± 1.2	5.1 ± 0.9	0.809
1 year	4.8 ± 1.1	5.4 ± 1.0	0.041
IKDC score			
Baseline	37.1 ± 7.8	39.2 ± 6.3	0.256
1 month	55.5 ± 8.2	62.7 ± 8.0	0.001
3 months	61.1 ± 8.0	65.5 ± 6.6	0.025
6 months	64.6 ± 6.1	64.9 ± 7.2	0.893
1 year	66.0 ± 5.2	62.0 ± 6.9	0.014
Lysholm score			
Baseline	54.4 ± 6.3	55.2 ± 5.7	0.640
1 month	68.6 ± 6.6	72.8 ± 7.5	0.024
3 months	72.7 ± 7.1	74.8 ± 7.5	0.260
6 months	76.7 ± 6.8	74.6 ± 5.7	0.199
1 year	77.6 ± 6.3	73.9 ± 5.9	0.020

Data are expressed as mean ± standard deviation. *MSC* Mesenchymal stem cell, *HA* Hyaluronic acid, *VAS* Visual analog scale, *IKDC* International Knee Documentation Committee

### Radiological outcomes

**Fig. 3:**
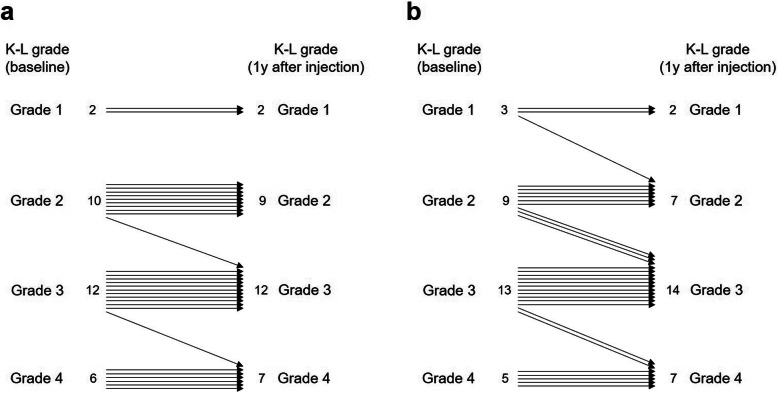
Changes in Kellgren-Lawrence (K-L) grades from baseline to 1-year post-injection in MSC **a** and HA **b** groups

**Fig. 4:**
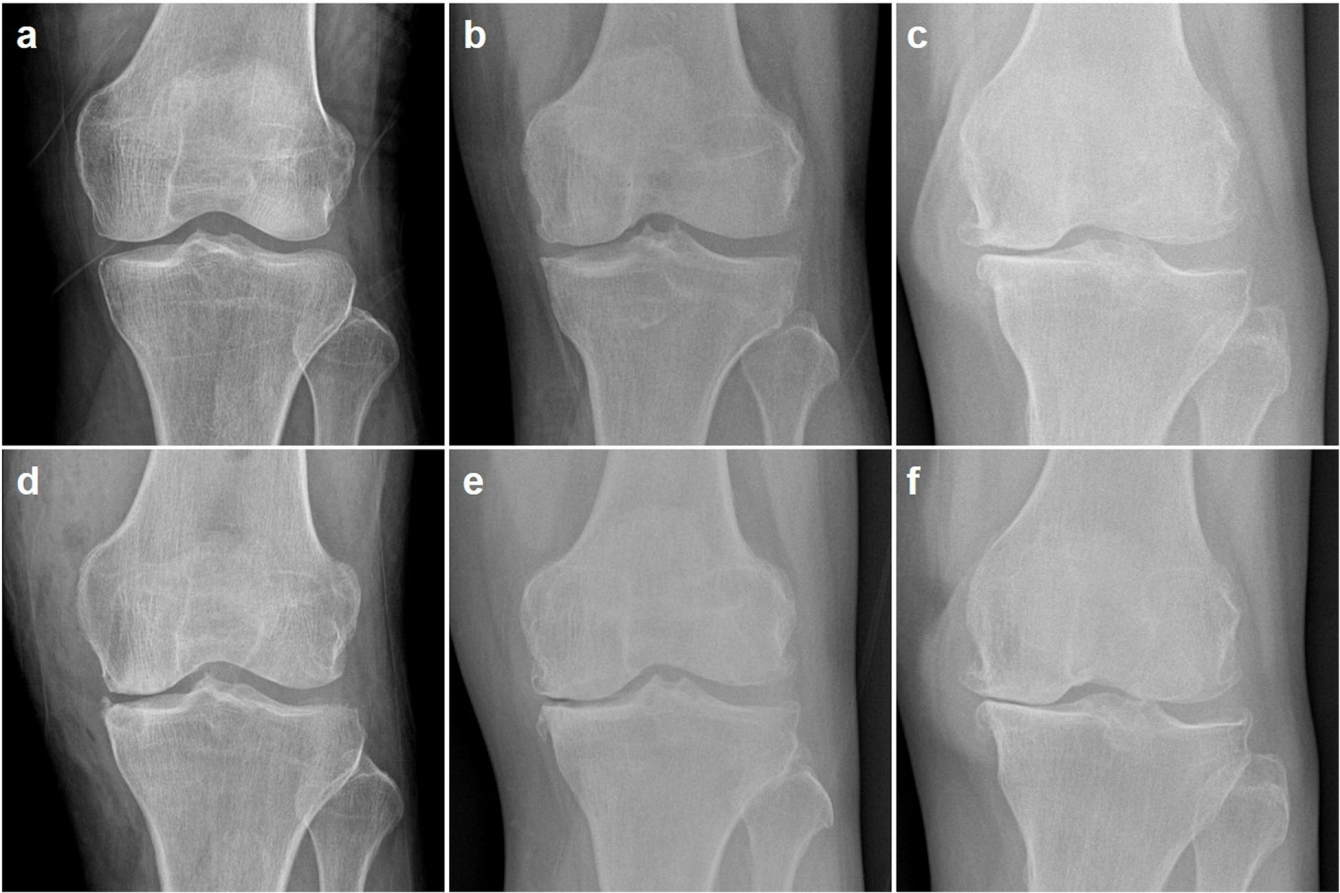
Radiographs showing the weight-bearing anterior-posterior (AP) views at baseline (**a**–**c**) and 1 year after injection (**d**–**f**). Radiographs of a 59-year-old man revealing a progression of knee osteoarthritis (OA) from Kellgren-Lawrence (K-L) grade 1 at baseline (**a**) to K-L grade 2 at 1 year after hyaluronic acid (HA) injection (**d**). Radiographs of a 64-year-old woman demonstrating an advancement of knee OA from K-L grade 2 at baseline (**b**) to K-L grade 3 at 1 year after mesenchymal stem cell (MSC) injection (**e**). Radiographs of a 62-year-old woman depicting a deterioration of knee OA from K-L grade 3 at baseline (**c**) deteriorating to K-L grade 4 at 1 year after HA injection (**f**)

According to the K-L grading system, a majority of the affected knees were categorized as grade 2 or 3 before injection (73.3% in both groups; Table 1), and there was no significant difference in K-L grade between the groups (*P* = 0.769; Table 1). The K-L grade worsened in a few patients (Figs. 3 and 4) especially in the HA group (6.67% in MSC group and 20% in HA group), and the majority of the knees were categorized as grade 3 or 4 at 1-year post-treatment (MSC group: 63.3%, HA group: 70.0%). However, there was no significant difference in K-L grade between the groups at 1-year post-injection (*P* = 0.742). We compared the preoperative and postoperative clinical outcomes according to the presence of radiological worsening in both groups, and found no significant differences (Table 3).

**Table 3:** Comparison of preoperative and postoperative clinical outcomes according to the presence of radiological worsening in both groups

	MSC			HA		
	Radiological worsening			Radiological worsening		
	Presence (*n* = 2)	Absence (*n* = 28)	*P* value^a^	Presence (*n* = 6)	Absence (*n* = 24)	*P* value^a^
VAS						
Baseline	8.0 ± 1.4	8.4 ± 1.1	0.604	8.5 ± 1.0	8.0 ± 1.1	0.359
1 year	5.5 ± 2.1	4.8 ± 1.1	0.372	5.7 ± 1.0	5.3 ± 1.0	0.404
*P* value^b^	< 0.001	< 0.001		< 0.001	< 0.001	
IKDC score						
Baseline	42.0 ± 11.3	36.7 ± 7.7	0.366	35.5 ± 4.6	40.1 ± 6.4	0.110
1 year	68.0 ± 8.5	65.9 ± 5.1	0.588	60.3 ± 6.1	62.5 ± 7.2	0.512
*P* value^b^	< 0.001	< 0.001		< 0.001	< 0.001	
Lysholm score						
Baseline	57.5 ± 10.6	54.2 ± 6.2	0.489	51.7 ± 4.2	56.0 ± 7.8	0.095
1 year	78.0 ± 11.3	77.6 ± 6.1	0.927	70.8 ± 5.8	74.6 ± 5.7	0.160
*P* value^b^	< 0.001	< 0.001		< 0.001	< 0.001	

Data are expressed as mean ± standard deviation. *MSC* Mesenchymal stem cell, *HA* Hyaluronic acid, *VAS* Visual analog scale, *IKDC* International Knee Documentation Committee

^a^Mann–Whitney U test

^b^Wilcoxon signed-rank test for comparison of clinical outcomes at baseline versus I year after the injection

## Discussion

To date, no studies have compared between adipose-derived MSC and HA. The identification of changes in clinical outcomes following MSC injection and the comparison to those observed following HA injection would provide patients with more accurate expectations of this treatment. To our knowledge, this is the first matched-pair study comparing the outcomes of an intra-articular injection of adipose-derived MSCs versus HA for the treatment of knee OA. Principally, both MSCs and HA resulted in clinical improvements. However, improvements were only continual and significant in the MSC group throughout the follow-up period. While the clinical outcomes were significantly enhanced and superior in the HA group until the 3-months follow-up, the improved conditions gradually reverted thereafter. Ultimately, MSC treatment surpassed HA treatment in begetting better clinical outcomes at the 1-year follow-up. On the other hand, the radiological outcomes of most patients remained unchanged in terms of K-L grading. Nevertheless, there were more patients in the HA group that showed signs of deterioration with K-L grade progression as compared to those in the MSC group, albeit no statistical significance.

Although the exact mechanism of HA in knee OA has not been elucidated, its clinical effects are probably mediated by several factors. Clinical studies revealed that pain relief may be attributed to the effects of HA on nerve impulses and sensitivities; HA reduces the activity of pain-related primary afferents by coating the pain receptors in the synovial tissues, and perhaps also traps molecules involved in pain signaling [44]. Several in vitro studies also indicated that HA administration can enhance the synthesis of extracellular matrix (ECM) proteins, which is helpful for restoring the cartilage matrix in diseased articular cartilage in OA-affected knee joints [24, 26, 48, 50]. Furthermore, in vivo studies [1, 39] demonstrated the ability of HA to prevent the release of ECM proteins from the cartilage matrix into joint space. Some studies also suggested that HA can suppress the production and activity of proinflammatory mediators and proteases, and alter the function of certain immune cells [15, 54]. Histological evidence showed that HA prevents cartilage degradation and may even promote its regeneration [17]. Collectively, HA seems to function mainly by reducing pain transmission and blunting inflammatory cascade. and stimulating synthesis and deposition of ECM molecules which are suppressed in implicated OA joints [40]. Similar to HA, MSCs have been reported to alleviate OA joint degeneration by improving the local microenvironment, immune-regulation, and anti-inflammatory biological activities through the secretion of exosomes, growth factors, cytokines, anti-inflammatory factors, and other bioactive molecules [59]. MSCs have also been shown to promote the proliferation of a pool of endogenous cells and contribute to chondrogenesis by renewing the ECM and synthesizing type II collagen [64]. Despite the capability of MSCs to differentiate into mesodermal cell lineages including cartilage, initial regenerative claims concerning therapeutic effects in OA have been revised due to recent evidence suggesting that paracrine and anti-inflammatory actions are crucial for tissue-restoring effects of MSC treatments [58]. Therefore, we investigated and compared the clinical outcomes over the 1-year follow-up period after intra-articular injections of either MSCs or HA. We speculate that the short-term clinical outcomes were mainly affected by the enhancement of the microenvironment, immune-regulation and anti-inflammatory effects rather than cartilage regeneration itself, because cartilage regeneration would probably require a longer time to occur or would not happen even at longer follow up after MSC injections as described in several previous studies [18, 33].

The efficacy of HA injections on pain relief and joint function restoration has been evaluated in numerous studies [10, 19, 26, 40, 41, 51], and recent systematic reviews have shown that improvements in pain intensity and functional outcomes were likely to take effect in 4–8 weeks and could last up to 6 months [3, 47]. Moreover, Suppan et al. [51] suggested that HA efficacy was sustainable for ≥1 year. Our findings were consistent with previously reported action duration of intra-articular injections of HA for knee OA. While the clinical outcomes in the HA group significantly improved only until 3 months and gradually reduced thereafter (Table 2 and Fig. 2), the post-treatment scores were still significantly better than the baseline scores; this implied that HA efficacy lasted for ≥1 year. Interestingly, a greater improvement in clinical outcomes was noted in the HA group in comparison to the MSC group for the first 3 months (Table 2). We speculate that these results were due to the antinociceptive effect of HA. Boettger et al. [7] reported that a single intra-articular injection of HA could alleviate pain by more than 50% in comparison to saline in a bradykinin/prostaglandin E2 animal model and the pain responses lasted for ≥56 days after administration. In addition, HA exposure has been shown to decrease arachidonic acid secretion from the fibroblasts isolated from patients with knee OA under bradykinin stimulation or calcium ionophore induction [53], which contributes to its antinociceptive effects. Furthermore, a recent in vitro study demonstrated that the stimulation of κ opioid receptors by HA [63]. Therefore, the superior clinical outcomes in the HA group for the first three-month scan be explained by these antinociceptive effects of HA. A previous meta-analysis suggested that intra-articular MSC injections could significantly improve pain scores for knee OA, which indicated that MSCs can be used for pain relief in short-term follow-up [27]. The analgesic effect of MSCs was also demonstrated by Orozco et al. [42] They evaluated the pain, disability, and quality of life after an intra-articular injection of bone marrow-derived MSCs in twelve patients with knee OA and found that the patients exhibited rapid and progressive improvement in algofunctional indices of up to 65–78% by 1 year post-treatment. They also reported a rather quick onset of analgesic effect, with more than 50% of the maximum improvement attained by the third month., but a tapered effect subsequently to reach the maximum effect at the 1-year follow-up. Similar findings were observed in our study; the mean VAS score in the MSC group gradually improved to 4.8 points at the 1-year follow-up, which was similar with the mean VAS score in the HA group at the 1-month follow-up (4.7 points). It should be noted that the improved clinical outcomes in the HA group deteriorated at some point during the follow-up period, while a steady improvement in clinical outcomes was achieved in the MSC group. Moreover, a greater improvement was eventually achieved at the 1-year follow-up in the MSC group than in the HA group (Table 2). Taken together, we consider that MSCs has a superior longevity to HA for its therapeutic effects especially in pain relief. In addition to the analgesic effects, a systemic review and meta-analysis proposed that MSC injection could be potentially efficacious not only for decreasing pain but also for improving physical function in patients with knee OA [61]. Another meta-analysis also demonstrated that the use of a recommended concentration of MSCs may result in favorable clinical outcomes; however, current evidence does not support the use of intra-articular injections of MSCs for cartilage repair in knee OA [28]. Here, we focused on investigating the clinical outcomes without evaluating the cartilage repair status due to a lack of evaluation tools. Given that no similar studies have been published, we believe that our findings are valuable in comparing the outcomes of MSCs versus HA treatments for knee OA.

Literature reviews revealed that the progression of knee OA is natural and inevitable [12, 36]. Several studies estimated the narrowing of joint space in patients suffering from knee OA to be 0.1–0.3 mm/year [6, 36]. A study involving 869 patients with knee OA revealed K-L grade worsening in 3% males and 4% females each year [12]. Herein, the K-L grade exacerbated in a few patients at 1-year post-injection, especially those in the HA group (Figs. 3 and 4). Although no statistically significant difference was found in the K-L grades between the two treatment groups at the 1-year follow-up (*P* = 0.742), we hypothesize that MSCs could possibly retard the progression of arthritic changes to a larger extent as compared to HA. However, the follow-up period of our study was relatively short (mean; MSC group: 14.2 months, HA group: 15.2 months). Therefore, long-term evaluations are required to investigate the effects of MSCs or HA on the progression of knee OA.

There are several limitations in the present study. First, we were unable to conduct this study in a blinded or randomized manner, and the data were collected retrospectively. However, case-matching according to gender and age in combination with strict inclusion and exclusion criteria were set to achieve a relatively homogeneous distribution of all the parameters that can potentially influence the post-treatment clinical outcomes. Therefore, we believe that these data are valuable in comparing the outcomes of MSCs versus HA in treating knee OA. Second, only VAS, IKDC, and Lysholm scores were considered for clinical evaluation and K-L grade was assessed for radiological evaluation. Other more specific OA-related scores such as Knee Injury and Osteoarthritis Outcome Score [46] or the Western Ontario and McMaster University Osteoarthritis Index [5] would have been more suitable. In addition, it is also important to examine the morphological changes of the articular cartilage for a more precise therapeutic evaluation. Hence, follow-up MRI or diagnostic and/or therapeutic arthroscopy is needed to analyze the association of MSC or HA injection and the mechanical properties and biological functions of the regenerative cartilage. Third, the optimal number of MSCs and dosage of HA remain unknown. Presently, low (0.5 × 10^6^–1 × 10^6^ Da), intermediate (2 × 10^6^ Da), or high (6 × 10^6^ Da) molecular weights (MW) of HA are marketed; it is recommended to administer the low and intermediate MW of HA weekly for three to five doses, and the high MW of HA as a single larger dose [51]. In this study, high MW of HA was used. However, further investigation is required to study the volume effect of single larger dose versus multiple smaller doses of intra-articular injection of high MW of HA in achieving maximum therapeutic effects in knee OA. Furthermore, studies are required to determine the optimal number of MSCs to be used for better clinical outcomes in the treatment of knee OA. In addition, an arthrocentesis was performed to eliminate a knee effusion before MSC or HA were administered. However, the effusion, especially requiring arthrocentesis, can deeply affect the injection results. The future study investigating the effect of effusion on the injection results is required for more precise comparing the outcomes of MSCs versus HA.

## Conclusion

This study showed that intra-articular injections of MSCs and HA improved the clinical outcomes of patients suffering from knee OA, without severe adverse effects, throughout the short-term follow-up period. Although more pronounced clinical improvements were evident in the HA group during the early follow-up periods, the MSC group eventually exhibited superior clinical improvements at the 1-year follow-up. However, a larger sample size and long-term prospective randomized studies are needed to validate our findings.

## Data Availability

The datasets used and/or analysed during the current study are available from the corresponding author on reasonable request.
